# The emerging clinical relevance of cell-free DNA in lupus: from mechanistic insights to therapeutic opportunities

**DOI:** 10.3389/fimmu.2026.1824772

**Published:** 2026-06-23

**Authors:** Emily Jones, Kiki Cano-Gamez

**Affiliations:** Department of Clinical and Biomedical Sciences, University of Exeter, Exeter, United Kingdom

**Keywords:** anti-dsDNA antibodies, biomarkers, cell-free DNA (cfDNA), epigenomics, fragmentomics, plasmacytoid dendritic cell (PDC), systemic lupus erythematosus (SLE), type I IFN

## Abstract

Systemic lupus erythematosus (SLE) is a complex autoimmune disease often characterised by dysregulated immune responses to self-DNA. Emerging evidence highlights the role of cell-free DNA (cfDNA) in promoting immune activation in SLE. Cell-free DNA is released primarily during cell death and is cleared by nucleases such as DNASE1L3. This review explores how molecular characteristics of cfDNA, such as fragment length, end motifs, jaggedness, and epigenetic modifications, serve as biomarkers of disease activity and may contribute to pathogenesis. The review then explains how deficiencies in DNASE1L3 alter cfDNA fragmentation, increase immunogenic potential, and enhance type I interferon signalling via plasmacytoid dendritic cells, driving B-cell differentiation and anti-double-stranded DNA (anti-dsDNA) antibody production. We also discuss how autoantibodies can cross-react to neutralise DNASE1L3, impeding DNA clearance and exacerbating inflammation. We conclude by highlighting how these findings underpin the dual role of cfDNA as both a disease mediator and a diagnostic tool and support the ongoing development of IFN-I and pDC-targeting therapies, which show promising clinical relevance. Moreover, we discuss how large-scale validation and standardisation are essential for translating these molecular insights into precision medicine for diverse SLE populations.

## 1 Introduction

Systemic lupus erythematosus (SLE) is a complex and systemic autoimmune disease which can affect a variety of organs such as the skin, joints, and kidneys (1). SLE arises from complex interactions between polygenic susceptibility, environmental triggers, and hormonal influences. It predominantly affects women of childbearing age, with a relapsing and remitting course marked by disease flares and periods of remission (1, 2). Disease activity is measured using the SLE Disease Activity Index (SLEDAI), and disease management typically encompasses the use of immunosuppressive and immunomodulatory therapies to target inflammation, prevent organ damage, and reduce flare frequency (3).

SLE is thought to be caused by the immune system mounting a response against healthy tissue components, including self-DNA. This leads to widespread inflammation and production of autoantibodies, which results in secondary tissue damage (1). An emerging area of research in SLE focuses on cell-free DNA (cfDNA), a collection of short DNA fragments released into the bloodstream via active secretion, as well as through various cell death pathways which link to efferocytosis (i.e., the clearance of cellular debris by mononuclear phagocytes). Cell-free DNA is often altered during SLE and can be leveraged as a tool for understanding and monitoring disease (4–7). While aberrant cfDNA is not an absolute feature of SLE, it is a common hallmark of disease. This is thought to relate to impaired efferocytosis, likely due to the presence of anti-nuclear autoantibodies and complement deficiencies. For example, the complement protein C1q usually promotes removal of apoptotic bodies and immune complexes. However, patients with C1q deficiencies develop autoantibodies and a lupus-like phenotype (1). Impaired removal of cellular debris can cause apoptotic cells to progress to secondary necrosis, further altering the release and characteristics of circulating chromatin (8). This results in a set of observable and measurable alterations which can be detected in cfDNA.

The study of cfDNA in SLE is a promising area of research, particularly in light of recent advances in the development of cfDNA-based diagnostics for other conditions, including pregnancy, cancer, infectious diseases, and organ transplantation (9–11), which highlight the potential of cfDNA as a versatile biomarker. The study of circulating cfDNA usually includes examination of structural features such as fragment size, end motifs, and chemical modifications like DNA methylation (9). These features contain information about the origin of cfDNA, as well as the processes involved in its fragmentation (12). In lupus, particularly in patients with monogenic forms of the disease, cfDNA displays abnormalities which indicate disrupted clearance (13). For example, whilst in healthy individuals the nuclease DNAse I-like 3 (DNASE1L3) facilitates most of the cfDNA clearance required to evade autoimmune responses (alongside supplementary clearance pathways like uptake by phagocytes and platelets, and renal filtration) (14), rare variants in the *DNASE1L3* gene (e.g., an Arg206Cys substitution) have been observed in patients with monogenic SLE (15). These variants are thought to cause disease by reducing DNASE1L3 activity, and thus cfDNA clearance (15). In addition to DNASE1L3, numerous other nucleases and genes are thought to contribute to SLE pathogenesis (16), making this a key area of investigation.

Beyond their biomarker potential, the aberrant cfDNA fragments observed in SLE patients can also trigger type I interferon (IFN-I) responses and stimulate the production of anti-double-stranded DNA (anti-dsDNA) antibodies, both of which are key drivers of autoimmunity (17–20). Understanding how cfDNA shapes immune responses in SLE could provide insights into disease mechanisms and potential novel treatments.

Thus, here we present a comprehensive review of published evidence that cfDNA features can be used as biomarkers for SLE monitoring. Moreover, we investigate how the disruption of cfDNA biogenesis and clearance pathways contributes to fragmentomic and epigenomic alterations in cfDNA. Finally, we explore how alterations in cfDNA can lead to aberrant IFN signalling, production of anti-dsDNA autoantibodies, and generalised autoimmune reactions.

## 2 Molecular features of cfDNA as potential biomarkers in SLE

**Figure 1:**
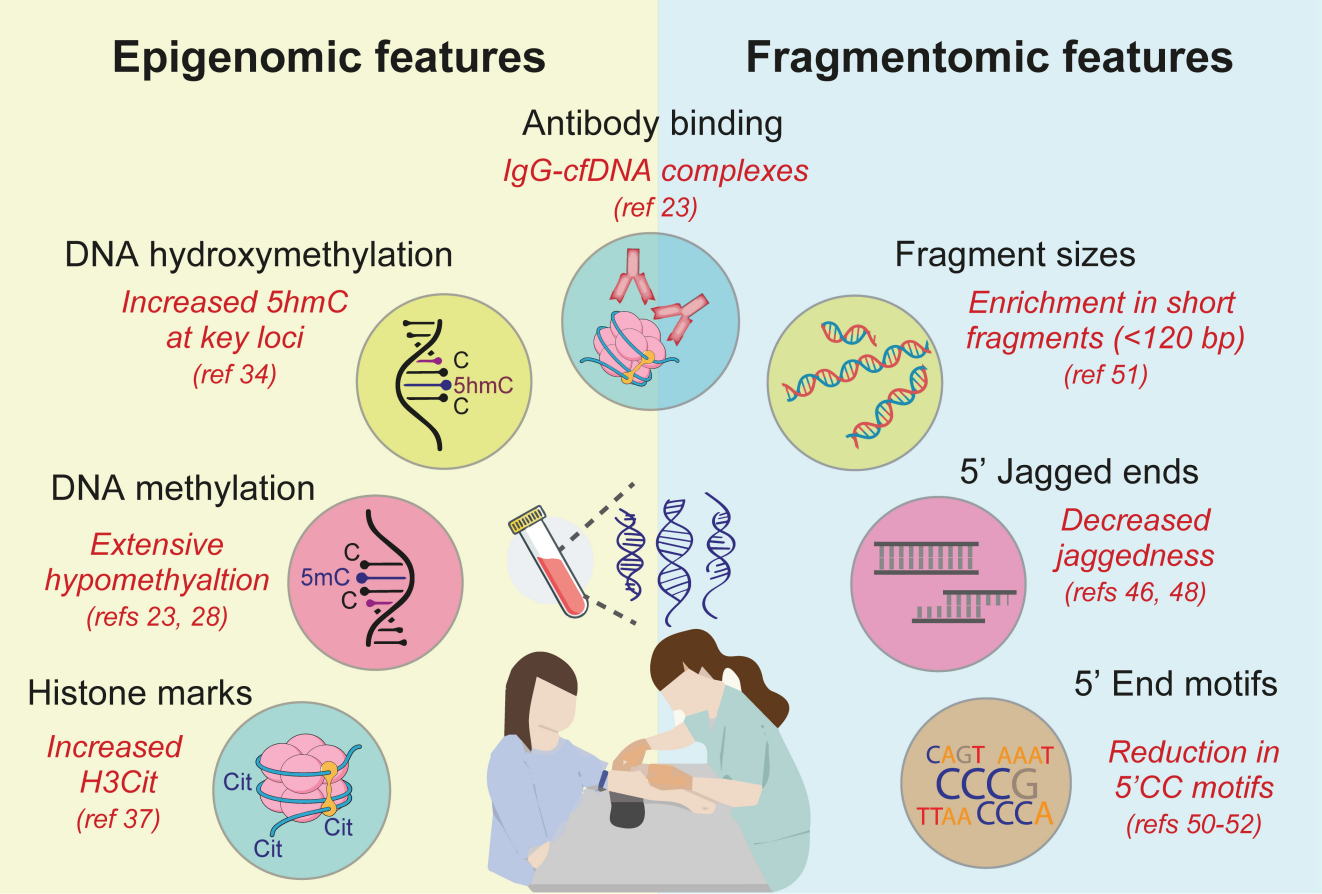
Candidate cfDNA-based lupus biomarkers. Schematic representation of key molecular features of cfDNA for which associations with SLE have been reported. Features are grouped into epigenomic (left-hand side) and fragmentomic (right-hand side) variables. Red text highlights published alterations seen in patients compared with controls. IgG, immunoglobulin G; H3Cit, citrullinated histone 3; 5hmC, 5-hydroxymethylcytosine.

Fragmentomics is the study of the fragmentation of cfDNA within extracellular fluids. DNA fragmentation is initiated during apoptosis by intracellular nucleases such as DNA fragmentation factor subunit beta (DFFB) and DNASE1L3 and proceeds after apoptosis in an extracellular manner, mediated by circulating nucleases such as DNASE1L3 and DNASE1 (21). Advances in cfDNA fragmentomics have uncovered a complex interplay between nuclease cutting and structural characteristics of cfDNA, providing novel insight into the pathophysiology of monogenic SLE. In parallel, studies of cfDNA epigenomics, particularly in patients carrying DNASE1L3 mutations, have revealed distinct co-existing patterns of dysregulated nuclease activity and epigenetic alterations, suggesting that nuclease cleavage is interlinked with epigenetic control mechanisms (22). In this section, we describe the most important epigenetic and fragmentomic characteristics of cfDNA, discuss how they relate to one another, and review how they can be used as candidate disease biomarkers. These characteristics, as well as their reported alterations during SLE, are summarised in **Figure 1**.

### 2.1 Candidate biomarkers based on cfDNA epigenetics

With the increased application of massively parallel genome and methylome sequencing technologies, numerous plasma DNA characteristics have been determined in SLE populations (23, 24). Key indications of biological processes underlying a disease state can be inferred from cfDNA monitoring, and these analyses can be obtained by minimally invasive techniques, as seen previously in oncology (25, 26).

Recently, studies have characterised cfDNA profiles with regard to cell type-specific and tissue-specific features. A pivotal finding of a 2014 sequencing analysis was the decreased methylation densities in the plasma DNA of active SLE (23). This study involved sampling 24 SLE patients and 10 healthy controls for methylation analysis, splitting patients using SLEDAI where inactive SLE was determined by an SLEDAI score ≤ 6 (n=15) and active SLE as SLEDAI > 6 (n=9). A strong correlation between hypomethylation and SLEDAI was determined. To better understand the observed correlation between hypomethylation and SLEDAI and anti-dsDNA antibody levels, a column-based protein G capture segregated immunoglobulin G (IgG)-bound and non-IgG-bound plasma DNA, finding that SLE individuals had higher IgG-bound cfDNA concentrations versus healthy individuals. Importantly, this IgG-bound DNA was both shorter and more hypomethylated compared with non-IgG-bound cfDNA, providing insight into key mechanisms behind the aberrant profiles of plasma DNA in lupus. In a 2023 mouse model knocking out DNASE1L3 in macrophages, total IgG levels were found to increase with age in the conditional knockout mice, suggesting the need to consider how environmental factors might affect clinical interpretation (27). Later in 2025, a genome-wide analysis leveraged multi-omics technologies to integrate observations of differentially expressed genes and differentially methylated probes from various cell environments, confirming reduced methylation in the cfDNA of lupus nephritis patients versus healthy controls (28).

While most studies have primarily focused on DNA methylation (29, 30), there is increasing evidence that different molecular characteristics of cfDNA are interrelated. For example, a 2023 study identified structural features of cfDNA (i.e., nucleosome footprints, tissue-specific preferred end motifs, and regulatory elements), and found DNA methylation to have a regulatory role in fragmentation (31). In both DNASE1L3- and DNASE1-deficient mice, as well as in DNASE1L3-deficient humans, deficiency of DNASE1L3 resulted in hypomethylated cfDNA. In contrast, deficiency of DNASE1 resulted in hypermethylated cfDNA.

These observations prompt us to consider how methylation could affect cfDNA fragmentation profiles. Reduced DNA methylation has been seen to enhance nucleosome accessibility, which in turn could increase the surface area of cfDNA that can be cleaved by nucleases. This influences the fragmentation of cfDNA, leading to variability in fragment sizes and cleavage sites. This relationship is crucial to consider in the context of cfDNA-based diagnostics, given that SLE prognosis can be somewhat predicted based on cfDNA methylation and fragmentation patterns (32). The interplay between cfDNA methylation and fragmentation is further supported by research on colorectal cancer cell lines treated with a demethylating agent, which resulted in increased nucleosome accessibility and enhanced DNA fragmentation into cfDNA (33).

Another epigenetic mark relevant in SLE is 5-hydroxymethylcytosine (5hmC). Comparative analysis of peripheral blood samples from SLE patients or healthy controls enabled investigation into the role of 5hmC modifications on plasma DNA. By using genome-wide hydroxymethylated DNA immunoprecipitation (hMeDIP) coupled with microarray chip analysis, a technique used to measure the distribution of 5hmC across the genome, this study demonstrated significant alterations of 5hmC in SLE samples (34). In particular, *TREX1, CDKN1A*, and *CDKN1B* were found to exhibit the most significant increase in 5hmC levels. While these observations come from whole blood and have not been directly tested in cfDNA, they support the use of differentially hydroxymethylated DNA sites as biomarkers of SLE. Since methylation markers remain present after fragmentation and provide tissue-specific insight, cfDNA could act as a candidate biomarker. This is in line with the clinical use of methylation within cfDNA-based liquid biopsy technologies in applications like oncology (35).

There is developing evidence that histone marks are also detectable in plasma cell-free nucleosomes and that they could provide information about the pathological processes operating within the tissues of origin of cfDNA (36). A 2025 comparative study focused on citrullinated histone H3 (H3Cit), a key post-translational modification which often marks neutrophil extracellular traps (NETs), and found elevated H3Cit in SLE patients versus healthy controls (37). Interestingly, H3Cit was significantly higher in SLE compared with other autoimmune disease such as rheumatoid arthritis, psoriatic arthritis, and axial spondyloarthritis. Although cfDNA is only partly derived from NETs and thus this evidence is limited to demonstrating NET-derived chromatin release, elevated H3Cit in SLE could reflect inflammation and vascular damage in a clinical setting. Post-translational modifications in NET-derived histones might also provoke certain autoantibodies to target the associated DNA, and simultaneously amplify inflammatory pathways (38). It has become imperative that future research should aim to measure other histone modifications within the cfDNA context in SLE.

Histone modifications reflect the regulatory events happening within DNA, for example in terms of RNA polymerase activity and non-coding regions, like promoters and enhancers, which can be compared against expected activity in healthy tissues (39). When cfDNA is in circulation, it retains elements of its original chromatin structure such as post-translational modifications in histones. These histone modifications can be used to trace cfDNA back to the dying cells from which it was derived. One study validated the use of blood samples to process cell-of-origin information from plasma cfDNA samples, utilising cell-free chromatin immunoprecipitation followed by sequencing (cfChIP-seq) with low sequencing depth (40). Since cfDNA has a limited half-life (between 15 min and 2 h), any tissue-specific information derived from unique methylation and histone modifications is thought to be indicative of events that occurred close to the time of sampling (41). An exact estimation of plasma DNA half-life was first attempted using intravenous injection of radiolabelled DNA in mice. This study observed full clearance of the radiolabelled mononucleosomal particles within 20 min, which was later reproduced in a separate experiment, showing 50% clearance of radiolabelled chromatin after 1 min and 90% clearance after 20 min (42–44). This supports the use of cfDNA as a near real-time biomarker of cell death.

Thus, epigenetic modifications are directly applicable for monitoring disease progression, particularly the dynamics of SLE tissue damage. Third-generation sequencing technologies like PacBio single-molecule real-time (SMRT) sequencing and Oxford Nanopore Technologies sequencing have now emerged to detect long cfDNA fragments (up to thousands of base pairs in length); these technologies will enable better inference of single-molecule methylation patterns and tissue-of-origin in future SLE research, although limitations have arisen, including the bias to overrepresent longer DNA fragments (45).

### 2.2 Candidate biomarkers based on cfDNA fragmentation

Recent progress has moved the field of cfDNA technology to include biofluid-specific analysis, long-read sequencing, single-stranded library preparation, and machine-learning-driven cfDNA analysis, which promise an exciting route to developing personalised medicine approaches in both SLE monitoring and early cancer detection (24).

Jagged ends, defined as double-stranded plasma DNA containing single-stranded ends, are a structural component suggested to indicate the preferential ranking of specific nuclease activity, and which may reflect NETosis (i.e., neutrophil cell death accompanied by release of NETs) as a potential mode of cell death (46, 47). Jaggedness can be assessed indirectly based on the lack of a DNA methylation signal at 5′ jagged ends Jaggedness can be assessed indirectly based on the seeming lack of DNA methylation at 5' jagged ends, which are synthetically converted to blunt ends during the end-repair step of library preparation (46). A recent study applied this procedure to plasma samples from mouse models lacking specific nucleases (46). This revealed that the three predominant nucleases known to be involved in cfDNA generation (DNASE1, DFFB, and DNASE1L3) are associated with different proportions of 5′ jagged ends. For example, whilst knocking out the *Dnase1* gene reduced jaggedness, silencing *Dnase1l3* enhanced jaggedness. However, a contradictory study reported a significant reduction in jaggedness upon deletion of *Dnase1l3*, which was supported by observations from human patients deficient in DNASE1L3 and presenting with familial SLE (48). The observed reduction in jaggedness from *Dnase1l3* silencing seemed to specifically affect cfDNA fragments composed of multiple nucleosomes (i.e., of larger molecular size), suggesting a potential cause for the observed discrepancies between studies. It is also critical to consider that technologies have recently evolved to single-stranded and end-preserving libraries, capturing both 5′ and 3′ end motifs, and this is being adopted across various diseases. For example, a 2026 single-stranded library preparation was able to capture both 5′ and 3′ ends simultaneously whilst avoiding end-repair artefacts (49). This will be translated in future cfDNA fragmentomics research, where end-motif features of cfDNA have direct biomarker potential in the context of SLE.

Individuals with SLE who carry DNASE1L3 mutations have been shown to exhibit a reduction in the proportion of “CC” end motifs in cfDNA (50). This was validated by adeno-associated viral transduction of DNASE1L3 into DNASE1L3-deficient mice, which restored end-motif profiles to those observed in the plasma DNA of wild-type mice (51). Another study utilising deconvolutional analysis of end motifs described a set of cleavage patterns distinct to cfDNA which relate to differential nuclease cutting activity. These were labelled “founder” end motifs (F-profile) and were validated through sequential nuclease-knockout mouse models (52). This study identified an F-profile linked to DNASE1L3 function (F-profile I), which was significantly reduced in individuals with active SLE. Importantly, F-profile I was also negatively correlated with SLEDAI scores, highlighting its association with disease severity (52). It is worth highlighting that different fragmentomic features of cfDNA are interrelated. This is best illustrated by a study which integrated functional genomics data, reporting a strong association between nuclease preference, end motifs, and cfDNA size (31). These observations might provide insights into future biomarker discovery for estimating SLE prognosis.

Skewed cell-free DNA size-distribution profiles are also clinically significant within SLE, initially characterised in a 2014 high-resolution analysis (23). Multiple studies have observed a dominant peak at approximately 166 bp across cfDNA samples, with certain tissue of origin specificity (29–31). Despite this consistency in dominant peaks, aberrations in cfDNA size were found to be associated with DNASE1L3-deficiency in mice, giving rise to an increase in short DNA molecules below 120 bp. Interestingly, these short molecules are positively correlated with anti-DNA antibody levels and reduced jagged ends (51). It has been hypothesised that antibody binding (i.e., IgG) may protect short DNA fragments from degradation by nucleases (23). These findings showcase the poorly understood causal relationship between cfDNA fragmentation and autoimmunity. However, such structural observations alone are insufficient to fully explain the mechanisms underlying autoimmune pathogenesis. In the future, *in vitro* stimulation assays using synthetic cfDNA fragments with defined sizes and end structures could be employed to assess their effects on immune cell activation, whereas biochemical analyses may help elucidate how cfDNA interacts with nucleic acid sensors.

The molecular characteristics of cfDNA, namely, fragment length, end motifs, jaggedness, and epigenetic modifications, offer potential for non-invasive biomarkers to capture real-time molecular pathology in SLE. These features hold significant potential to enhance diagnosis via liquid biopsy, enable dynamic disease monitoring, and inform personalised therapeutic strategies. Nonetheless, their clinical utility remains limited by unresolved mechanistic uncertainties and the need for robust validation in larger human cohorts. Such work would enable us to move beyond the current standard of care for SLE monitoring, which relies heavily on clinical judgement and assays for anti-DNA antibodies and cytokines, towards the use of cfDNA to uncover variables such as tissue damage during disease flares (i.e., cfDNA tissue of origin) and real-time disease activity estimation through its molecular signatures.

## 3 Interactions between cfDNA and the immune system during SLE

Molecular features such as fragmentation, methylation status, and structural characteristics influence the immunogenic potential of cfDNA. Aberrant cfDNA can activate innate immune sensors, particularly in the context of nuclease deficiencies, leading to altered type I interferon (IFN-I) signalling. This provides a direct link between cfDNA structural dysregulation and the autoimmune responses characteristic of SLE, providing a mechanistic bridge to IFN induction and autoantibody production.

### 3.1 Cell-free DNA triggers aberrant type I IFN signalling

**Figure 2:**
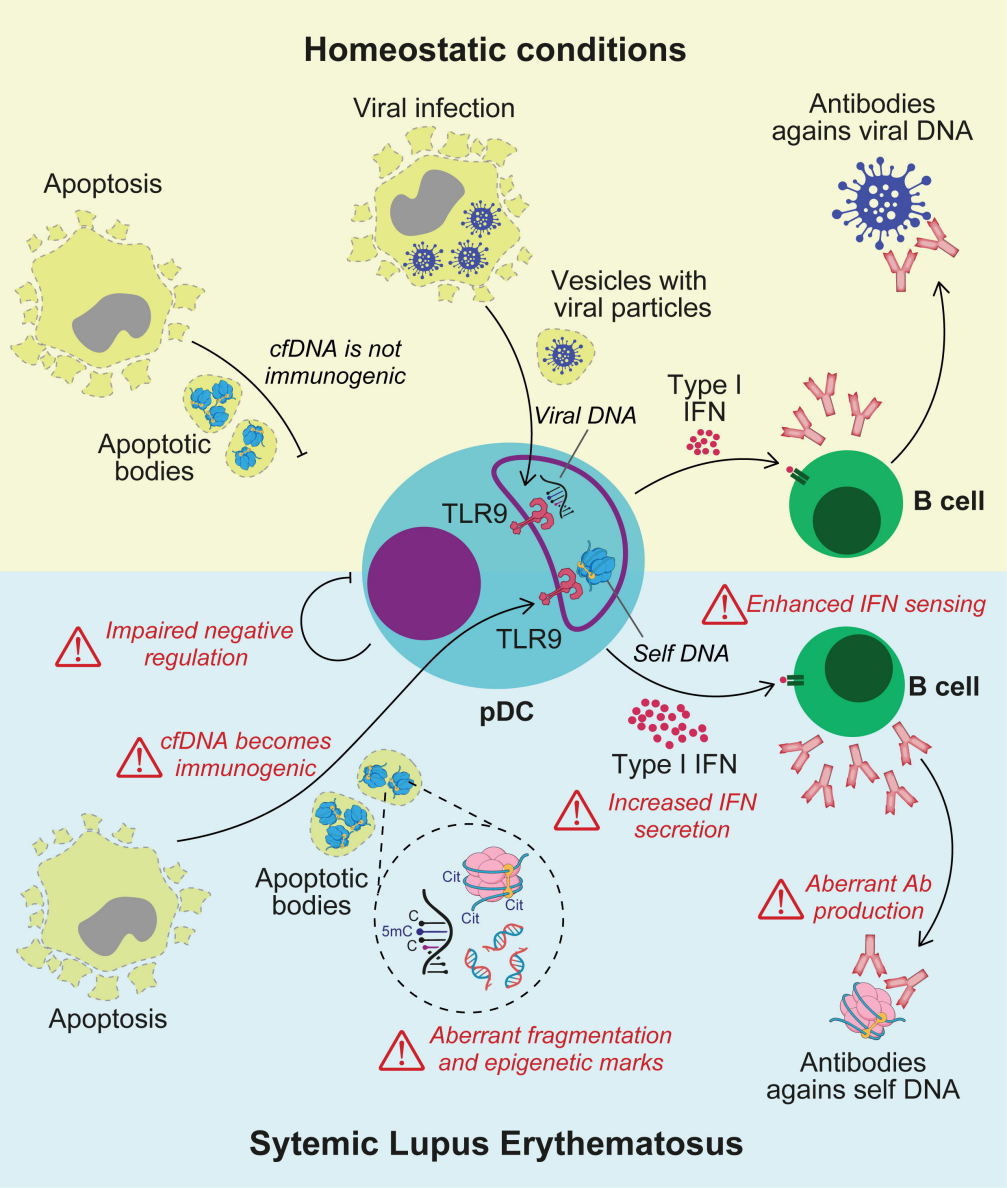
Aberrant activation of type I IFN signalling by cfDNA during SLE. Schematic representation of the molecular mechanisms thought to cause sustained type I IFN activity during SLE. Homeostatic conditions (top panel, yellow) are directly contrasted with disease conditions (bottom panel, blue). Key alterations observed in active SLE are highlighted in red and include (1) release of abnormal cfDNA during cell death (e.g., cfDNA displaying aberrant fragmentation or unconventional histone marks), which acts as an IFN inducer; (2) persistent activation of IFN-producing cells such as pDCs; (3) overproduction of type I IFNs, which amplifies signalling; (4) the presence of genetic variants which enhance the cellular response to type I IFNs in target cells (e.g., B cells); and (5) a defective negative feedback loop which fails to restrict IFN signalling, exacerbating damage. The cumulative effect of these alterations causes continuous IFN-I expression and results in the characteristic IFN signatured observed in SLE patients. IFN, interferon; pDC, plasmacytoid dendritic cell; TLR9, Toll-like receptor 9; Cit, citrullination; 5mC, 5-methycytosine; Ab, antibody.

Cytoplasmic DNA sensing pathways have been thought to contribute to autoimmunity via aberrant IFN signalling, which triggers an autoinflammatory cascade (53). Plasmacytoid dendritic cells (pDC) are a cell type generated in the bone marrow whose primary function is to detect pathogen-derived nucleic acids and to respond to them by producing IFN-I (54). However, continuous production of IFN-I by circulating pDCs is a known feature of SLE, often accompanied by increased expression of IFN-stimulated genes (ISGs), which manifests as an IFN signature (55, 56). Key mechanisms underlying the observed IFN signature in SLE are summarised in **Figure 2**, including (1) the presence of IFN inducers such as self-DNA released by dying cells; (2) the activation of other IFN-producing innate cells like monocytes; (3) the production of diverse IFNs to strengthen the response; (4) genetic predisposition to increased IFN production, and (5) a deficient negative feedback loop, by which individuals with SLE can no longer regulate the duration of IFN release or prevent secondary damage (53).

The IFN signalling alterations described above are intimately linked to cfDNA biology, as endosomal nucleic acid sensing can directly influence the activating threshold for release of IFN-I by pDCs. Toll-like receptors 7 (TLR7) and 9 (TLR9) have been associated with self-DNA recognition by pDCs (57). Thus, abnormalities in cfDNA profiles can trigger TLR7 (a single-stranded RNA sensor) and TLR9 (an unmethylated double-stranded DNA sensor), altering IFN activity and ultimately impacting immune function (58). TLR9 is especially sensitive to hypomethylated self-DNA as seen in lupus, and fragmented cfDNA also increases the accessibility of CpG motifs to TLR9 in endosomes. This is best illustrated by DNASE1L3 deficiencies, which have been associated with a transient increase in the level of ISGs in the blood (59, 60). This suggests that the interplay between DNASE1L3-deficiency and increased ISGs may be responsible for playing a stimulatory role in autoreactivity, which subsequently manifests as SLE.

These molecular alterations have consequences that extend beyond pDCs and into the other immune cells they interact with. For instance, anti-DNA autoreactivity is driven by B-cell differentiation into temporal plasmablasts, which is itself facilitated by pDCs, and thus linked to IFN-I signalling and endosomal TLR7 and TLR9 (61, 62). This is because pDCs express high levels of TLR7 and TLR9 and facilitate signalling through the MyD88 adaptor and CD32, coordinating the B-cell differentiation process (63). Thus, altered TLR signalling does not only affect pDCs but also can impact B cells and the autoantibody pool. We explore the link between these processes in the following section.

The cytosolic DNA sensor cyclic guanosine monophosphate-adenosine monophosphate synthase (cGAS) and its downstream signalling adaptor stimulator of interferon genes (STING) have also been described to elicit production of type I IFNs in response to aberrant cfDNA (64, 65). Evidence for the importance of cGAS-STING within SLE has been shown in numerous murine models with lupus-like presentation which have used loss-of-function mutations in *TREX1*, a gene encoding an exonuclease that digests circulating DNA particles. One study of the role of cGAS in autoimmune disease noted a decrease in tissue destruction and type I IFN production in double knockout *Trex1/cGAS* mice (66). These findings support a strong association between cfDNA, cGAS activation, and interferon-mediated pathology in SLE.

### 3.2 Aberrant IFN signalling promotes autoantibody production

The relationship between B-cell responses, anti-dsDNA antibodies, and IFN signalling is complex and highly regulated, as shown in **Figure 2**. In SLE, anti-dsDNA antibodies form immune complexes with endogenous circulating DNA. These complexes are delivered into pDCs, where they too elicit an IFN response (61). It has been suggested that particular genetic variants carried by SLE patients, such as *IRF5* and *IRF7* variants, could promote a more potent IFN response to these autoantibody complexes, consequently increasing IFN-I production (56). This explains how anti-dsDNA autoantibodies trigger the IFN pathway and exacerbate inflammation. However, it fails to explain how the activation of an IFN response to circulating nucleic acids results in the release of anti-dsDNA antibodies in the first place.

Previous studies on B-cell differentiation could shed light on this question. For example, Soni et al. report that IFN-I and associated pDCs facilitate the differentiation of self-DNA-reactive antibody-forming cells both *in vivo* and *in vitro* (57). The TLR9 DNA sensor in particular promotes these anti-dsDNA mechanisms and the development of SLE pathology, as demonstrated in DNASE1L3-deficient mice. These findings establish B-cell differentiation into antibody-forming cells as a key mechanism in the pathogenesis of SLE, revealing the complex role of pDCs, endosomal TLRs and IFN-I in autoreactivity (67). Similarly, nucleic acids from apoptotic neutrophils have also been shown to activate both innate and adaptive immune responses through TLRs (68, 69).

### 3.3 Emerging therapeutic strategies based on IFN modulation

Despite limited research on IFN signalling and autoreactivity mechanisms, some IFN-targeting therapies have succeeded into the clinical trial phase. In the context of SLE management, there has been considerable success in drugs blocking IFN-I signalling. This, along with the characterisation of tissue-specific autoinflammatory interferonopathies, establishes the potential for new targets to give rise to personalised treatment approaches (70). One novel IFN-targeting drug, anifrolumab, is a monoclonal antibody which blocks the IFN-I subunit 1. Anifrolumab has shown to induce a significant reduction in disease activity in SLE in randomised controlled Phase IIb and III trials (56, 71, 72). The advent of anifrolumab led to the discontinuation of sifalimumab, a previous drug used to target IFN-α in SLE, due to its superior efficacy (73, 74).

Similarly, TYK2 inhibitors, a drug family which functions by blocking tyrosine kinase 2 (TYK2), a kinase crucial for IFN-I signalling, have been included in an advanced Phase III trial for SLE treatment, following their approval for moderate-to-severe psoriasis, showing alternative methods of modulating the IFN pathway (75). Hence, IFN-blocking could uncover a new promise of precision medicine in rare monogenic disease when leveraged in parallel with real-time monitoring of tissue-specific features and organ inflammation.

More recently, pDCs have also emerged as a target for SLE therapy. One monoclonal antibody, VIB7734, has been shown to deplete pDCs in the setting of autoimmune disease, as evidenced in non-human primates (76). The results showed that VIB7734 can reduce symptom severity in lupus and found pDC-targeted therapies to be both feasible and effective in managing patients with autoimmune disease. However, this antibody has only been tested in two Phase I studies. A Bcl-2 inhibitor known as venetoclax has also been hypothesised to induce pDC apoptosis, contextualised by their innate dependence on BCL-2 for survival (77, 78). Nonetheless, this has only been studied in the context of pDC-targeting therapies for cancer (79). The effects of transient depletion of pDCs prior to SLE initiation have also been measured using lupus-prone mice in a 2014 study. This study found that pDC depletion impairs the differentiation and activation of adaptive immune cells and inhibits the expression of IFN-I induced genes (80). This combined evidence validates pDCs as a candidate target for future SLE therapies (61).

## 4 Cell-free DNA and anti-dsDNA antibodies in SLE

The immunostimulatory role of cfDNA in activating IFN signalling and promoting autoreactive B-cell responses directly influences the emergence of anti-dsDNA antibodies. Structural abnormalities in cfDNA, particularly those resulting from DNASE1L3 deficiency, can drive persistent interferon responses and facilitate loss of immune tolerance. Thus, understanding the interplay between cfDNA dysregulation and autoantibody development could offer critical insight into the complex pathogenesis of SLE.

### 4.1 Impact of DNASE1L3 activity on the autoantibody landscape

The pathological landscape of SLE is characterised by the presence of autoantibodies, particularly anti-dsDNA antibodies, which correlate with disease severity, most significantly in lupus nephritis (81). This relationship raises the question: how does DNASE1L3 deficiency impact the autoantibody landscape, particularly the emergence of anti-dsDNA antibodies?

In a recent study, more than 50% of sporadic SLE patients were observed to have attenuated DNASE1L3 function, which was linked to the manifestation of neutralising autoantibodies (82). Interestingly, it was recorded that these patients had circulating microparticles which contained longer polynucleosomal fragments of cfDNA (82). Polynucleosomal fragments can bind to autoantibodies with higher affinity than shorter nucleotide chains, which could explain how attenuated DNASE1L3 might bring about anti-dsDNA autoreactivity.

**Figure 3:**
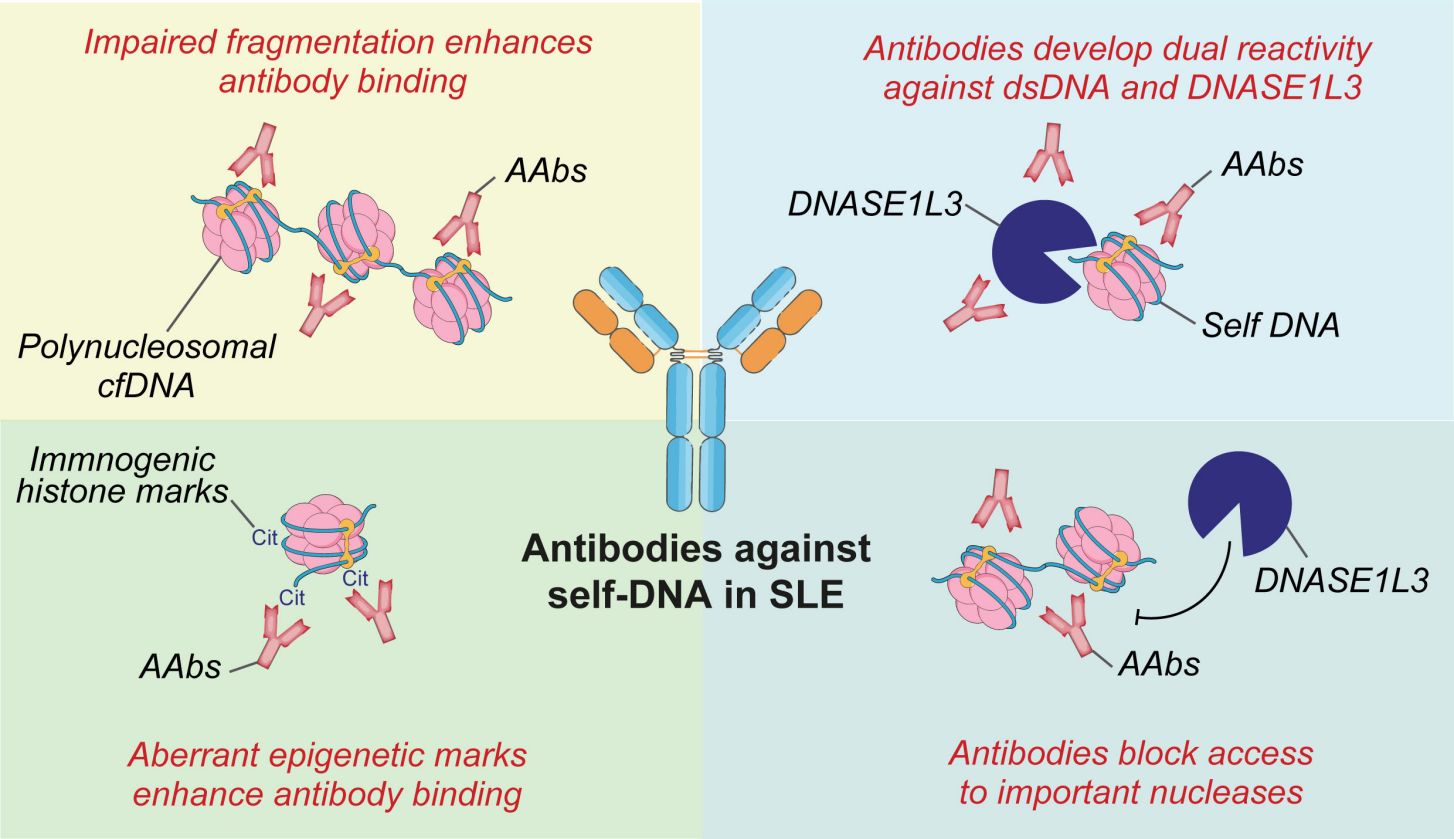
The crosstalk between anti-dsDNA antibodies and cfDNA in SLE. Diagram illustrating key features of autoantibodies against self-DNA reported in SLE patients. These include (1) antibodies developing dual reactivity to DNASE1L3 and dsDNA; (2) antibody–DNA complexes blocking access to key nucleases, thus disrupting cfDNA fragmentation; (3) autoantibodies against specific chromatin marks; and (4) autoantibodies binding more effectively to under-fragmented, polynucleosomal cfDNA fragments. AAbs, autoantibodies; dsDNA, double-stranded DNA.

Moreover, there is evidence of autoantibody-mediated impairment of DNASE1L3 activity as a facilitator of autoreactivity (82). This suggests that autoantibodies may not only be a consequence of DNASE1L3 deficiency but could also exacerbate its deficiency by impeding enzymatic activity, thereby creating a vicious cycle of autoimmunity (**Figure 3**). This highlights the complexity of genetic and immunological mechanisms in lupus pathology.

### 4.2 Crosstalk between anti-dsDNA antibodies and DNASE1L3

Affinity maturation describes the process by which B cells develop and secrete antibodies with a higher affinity for a specific antigen (83). Anti-dsDNA antibodies have been described as clinically heterogeneous, with variation stemming from their affinity maturation process during development (84). One study re-classified particular anti-dsDNA antibodies as anti-DNASE1L3 and considered that a high proportion of these expressed dual reactivity to both molecules (84). Significantly, the study found that even whilst having the affinity to bind to either DNASE1L3 or dsDNA, these particular antibodies typical of SLE pathology would preferentially interact with DNASE1L3. This might imply that antibodies initially target DNASE1L3 and evolve to express dual reactivity, which enhances their affinity and contributes to pathogenesis (**Figure 3**). This cross-reactivity can be attributed to structural similarities between the epitopes on dsDNA and DNASE1L3, allowing for a broader immune response against both self and foreign DNA (85). The significance of these dual reactive antibodies in monogenic SLE is further exemplified by various monoclonal antibody data showing increased pathogenicity of anti-DNASE1L3/dsDNA relative to anti-dsDNA antibodies alone (86).

The molecular characteristics of anti-dsDNA antibodies present a balance between specificity and affinity. The interplay between loss-of-function variants of DNASE1L3 and the autoantibody landscape in SLE points to a causal relationship where autoantibodies neutralise DNASE1L3 enzymatic activity, resulting in aberrant cfDNA accumulation and the formation of more pathogenic cross-reactive anti-dsDNA antibodies (**Figure 3**). This cyclical interaction underscores the need for thoughtful research into therapeutic strategies that could target these mechanisms as a way of managing SLE. Understanding these dynamics is crucial for developing interventions that can both protect and maintain the regulated balance of immune tolerance, thus mitigating the effects of autoimmunity.

## 5 Discussion

Novel findings in the fields of SLE and fragmentomics offer perspectives into how cfDNA contributes not only to immune activation but also to diagnostic and therapeutic opportunities. Fragmentomics has advanced our understanding of SLE pathogenesis by revealing how deficiencies in nucleases, such as DNASE1L3, alter DNA fragmentation patterns and promote autoimmune responses. These molecular disruptions in cfDNA clearance contribute directly to the activation of IFN-I pathways, the generation of anti-dsDNA antibodies, and the chronic inflammation that characterises SLE.

A critical insight from this review is the close association between specific cfDNA signatures, such as fragment length, end motifs, jaggedness, and methylation status. Aberrant fragmentation patterns, including the presence of short DNA fragments (<120 bp) and altered end motifs, have been shown to correlate with DNASE1L3 deficiency and heightened anti-DNA antibody production, alongside altered epigenetic modifications. These findings highlight the potential of cfDNA features as non-invasive biomarkers that can reflect ongoing immune activity and tissue damage in real time. As such, respective biomarkers could be integrated into standardised clinical care once sufficient data have been collated within diverse human populations.

The translational value of this research is considerable, as cfDNA analysis may support early diagnosis, monitoring of disease activity, and treatment stratification. For example, cfDNA profiles may help identify patients likely to respond to IFN-I blockade, as seen with anifrolumab, a monoclonal antibody targeting the IFN-I receptor that has shown efficacy in recent Phase III trials (87). Similarly, targeting pDCs may offer an additional route for disrupting the IFN-mediated autoimmune cascade (88, 89). These therapeutic developments could bring us closer to realising the promise of precision medicine in SLE.

Therapeutic advancement has been seen in a recent proof-of-concept study evaluating the use of an extracorporeal therapeutic apheresis device that selectively reduces the quantity of cfDNA and histone-containing NETs. This technology used beads coated with H1.3 to capture circulating DNA to directly remove pathogenic chromatin (90). After three apheresis sessions, cfDNA levels in circulation were near zero. This was observed in a single patient suffering with severe SLE (SLEDAI-2K= 32), resulting in a reduction in their SLEDAI-2K score to 12 immediately after therapy and to 6 at 3 months.

Moreover, the discovery of dual-reactive autoantibodies that bind both DNASE1L3 and dsDNA adds an additional layer of complexity to the immunopathology of SLE. These antibodies not only reflect immune dysregulation but may actively impair DNASE1L3 function, thereby perpetuating cfDNA accumulation and sustaining the autoimmune cycle. This self-reinforcing loop suggests a critical need for therapies that suppress immune responses, whilst also restoring physiological DNA clearance mechanisms.

Several key challenges remain within the advent of personalised SLE management. Much of the current evidence is based on small cohorts or animal models, and larger studies in diverse human populations are essential to validating cfDNA features as robust biomarkers. Standardisation of cfDNA extraction, sequencing, and analysis methods are also necessary to ensure reproducibility and clinical applicability. Furthermore, the temporal dynamics of cfDNA release and its immunological effects across disease stages are not yet fully understood for immediate integration of these approaches into standardised clinical care.

Future research should aim to integrate physiological cfDNA data with other molecular and clinical information, including transcriptomic and proteomic profiles. Longitudinal studies tracking cfDNA changes over time, especially during disease flares, remission, or treatment response, should be prioritised to unveil the full potential of cfDNA as a dynamic biomarker within extensive trials. Investigating how cfDNA characteristics differ across SLE subtypes, comorbidities, and genetic backgrounds may also reveal new pathophysiological mechanisms and therapeutic targets applicable to diverse populations.

To validate the clinical and biomarker development potential of cfDNA, large-scale multi-centre cohort studies should be conducted utilising clinical parameters like inflammation markers, symptom persistence, and disease severity scores, alongside metrics to measure organ involvement. These studies would provide necessary comparisons between the current standard of care and the use of cfDNA biomarkers to understand the clinical impact and cost effectiveness for policymakers. With regard to therapeutic innovation of pDC-targeted and IFN-targeted treatments, it would be highly beneficial to perform head-to-head trials comparing these approaches with existing lupus treatments such as biologics like belimumab. There could also be value in evaluating cfDNA dynamics pre- and post-administration of belimumab to monitor and improve clinical efficacy (91).

By decoding the structural and epigenetic basis of cfDNA, researchers and clinicians can gain valuable insights into the onset and progression of lupus, opening the door to earlier detection, more precise therapies, and better patient outcomes. This report lays the foundations for future investigations into the diagnostic and immunological potential of cfDNA in SLE and advocates for its integration into the broader landscape of personalised medicine.

## References

[1] Ameer MA Chaudhry H Mushtaq J Khan OS Babar M Hashim T . An overview of systemic lupus erythematosus (SLE) pathogenesis, classification, and management. Cureus. (2022) 14:1–16. doi: 10.7759/cureus.30330 36407159 PMC9662848

[2] Siegel CH Sammaritano LR . Systemic lupus erythematosus. JAMA. (2024) 331(17):1480–91. doi: 10.1001/jama.2024.2315 38587826

[3] Su X Yu H Lei Q Chen X Tong Y Zhang Z . Systemic lupus erythematosus: pathogenesis and targeted therapy. Mol BioMed. (2024) 5:54. doi: 10.1186/s43556-024-00217-8 39472388 PMC11522254

[4] Thierry AR . Circulating DNA fragmentomics and cancer screening. Cell Genom. (2023) 3:100242. doi: 10.1016/j.xgen.2022.100242 36777187 PMC9903826

[5] Helzer KT Sharifi MN Sperger JM Shi Y Annala M Mootsma ML . Fragmentomic analysis of circulating tumor DNA-targeted cancer panels. Ann Oncol. (2023) 34:813–25. doi: 10.1016/j.annonc.2023.06.001 37330052 PMC10527168

[6] Wang F Liu YJ Miao HB Chen Z . Clinical algorithm model based on cfDNA to predict SLE disease activity. Lupus. (2024) 33:145–54. doi: 10.1177/09612033231226314 38183242

[7] Li Y Ge F Liu C Pu W Lv W Zeng Z . Genome-wide characterization of extrachromosomal circular DNA in SLE and functional analysis reveal their association with apoptosis. Trans Res. (2024) 273:115–26. doi: 10.1016/j.trsl.2024.08.004 39173965

[8] Kenyon KD Cole C Crawford F Kappler JW Thurman JM Bratton DL . IgG autoantibodies against deposited C3 inhibit macrophage-mediated apoptotic cell engulfment in systemic autoimmunity. J Immunol. (2011) 187:2101–11. doi: 10.4049/jimmunol.1003468 21813769 PMC3159788

[9] Jin C Liu X Zheng W Su L Liu Y Guo X . Characterization of fragment sizes, copy number aberrations and 4-mer end motifs in cell-free DNA of hepatocellular carcinoma for enhanced liquid biopsy-based cancer detection. Mol Oncol. (2021) 15:2377–89. doi: 10.1002/1878-0261.13041 34133846 PMC8410516

[10] Lui YYN . Origin of plasma cell-free DNA after solid organ transplantation. Clin Chem. (2003) 49:495–6. doi: 10.1373/49.3.495 12600963

[11] Berzero G Pieri V Mortini P Filippi M Finocchiaro G . The coming of age of liquid biopsy in neuro-oncology. Brain. (2023) 146:4015–24. doi: 10.1093/brain/awad195 37289981 PMC10545511

[12] Grabuschnig S Bronkhorst AJ Holdenrieder S Rosales Rodriguez I Schliep KP Schwendenwein D . Putative origins of cell-free DNA in humans: A review of active and passive nucleic acid release mechanisms. Int J Mol Sci. (2020) 21:8062. doi: 10.3390/ijms21218062 33137955 PMC7662960

[13] Műzes G Bohusné Barta B Szabó O Horgas V Sipos F . Cell-free DNA in the pathogenesis and therapy of non-infectious inflammations and tumors. Biomedicines. (2022) 10:2853. doi: 10.3390/biomedicines10112853 36359370 PMC9687442

[14] Malki Y Zhou Q Jiang P Lo YMD . The comings and goings of cell-free DNA: Biological and clinical implications. Med. (2025) 7(2):100926. doi: 10.1016/j.medj.2025.100926 41274293

[15] Coke LN Wen H Comeau M Ghanem MH Shih A Metz CN . Arg206Cys substitution in DNASE1L3 causes a defect in DNASE1L3 protein secretion that confers risk of systemic lupus erythematosus. Ann Rheum Dis. (2021) 80:782–7. doi: 10.1136/annrheumdis-2020-218810 33455918 PMC8142439

[16] Moser KL Kelly JA Lessard CJ Harley JB . Recent insights into the genetic basis of systemic lupus erythematosus. Genes Immun. (2009) 10:373–9. doi: 10.1038/gene.2009.39 19440199 PMC3144759

[17] Kaan ED Brunekreef TE Drylewicz J van den Hoogen LL van der Linden M Leavis HL . Association of autoantibodies with the IFN signature and NETosis in patients with systemic lupus erythematosus. J Trans Autoimm. (2024) 9:100246–6. doi: 10.1016/j.jtauto.2024.100246 39027720 PMC11254743

[18] Dema B Charles N . Autoantibodies in SLE: Specificities, isotypes and receptors. Antibodies. (2016) 5:2. doi: 10.3390/antib5010002 31557984 PMC6698872

[19] Fillatreau S . Antibodies against type I IFN: The bad guys self-restrain in systemic lupus erythematosus. Cell Rep Med. (2023) 4:100903. doi: 10.1016/j.xcrm.2022.100903 36652912 PMC9873922

[20] Dai X Fan Y Zhao X . Systemic lupus erythematosus: updated insights on the pathogenesis, diagnosis, prevention and therapeutics. Sig Transduc Targ Ther. (2025) 10:102. doi: 10.1038/s41392-025-02168-0 40097390 PMC11914703

[21] Han DSC Ni M Chan RWY Chan VWH Lui KO Chiu RWK . The biology of cell-free DNA fragmentation and the roles of DNASE1, DNASE1L3, and DFFB. Am J Hum Genet. (2020) 106:202–14. doi: 10.1016/j.ajhg.2020.01.008 32004449 PMC7010979

[22] Zhou Q Kang G Jiang P Qiao R Lam WKJ Yu SCY . Epigenetic analysis of cell-free DNA by fragmentomic profiling. PNAS. (2022) 119:e2209852119. doi: 10.1073/pnas.2209852119 36288287 PMC9636966

[23] Chan RWY Jiang P Peng X Tam LS Liao GJW Li EKM . Plasma DNA aberrations in systemic lupus erythematosus revealed by genomic and methylomic sequencing. PNAS. (2014) 111:e5302–11. doi: 10.1073/pnas.1421126111 25427797 PMC4267379

[24] Swarup N Leung HY Choi I Aziz MA Cheng JC Wong DTW . Cell-free DNA: Features and attributes shaping the next frontier in liquid biopsy. Mol Diagn Ther. (2025) 29:277–90. doi: 10.1007/s40291-025-00773-x 40237938 PMC12062165

[25] Liskova A Samec M Koklesova L Giordano FA Kubatka P Golubnitschaja O . Liquid biopsy is instrumental for 3PM dimensional solutions in cancer management. J Clin Med. (2020) 9:2749. doi: 10.3390/jcm9092749 32854390 PMC7563444

[26] Batool SM Yekula A Khanna P Hsia T Gamblin AS Ekanayake E . The Liquid Biopsy Consortium: Challenges and opportunities for early cancer detection and monitoring. Cell Rep Med. (2023) 4:101198–8. doi: 10.1016/j.xcrm.2023.101198 37716353 PMC10591039

[27] Engavale M Hernandez CJ Infante A LeRoith T Radovan E Evans L . Deficiency of macrophage-derived Dnase1L3 causes lupus-like phenotypes in mice. J Leuk Biol. (2023) 114:547–56. doi: 10.1093/jleuko/qiad115 37804110 PMC10843819

[28] Liu Y Wang F Zhang Y Pei Z Chen Z . Plasma cell-free DNA genome-wide methylation profiling enables detection and activity assessment in systemic lupus erythematosus. Front Immunol. (2025) 16. doi: 10.3389/fimmu.2025.1721954 41488623 PMC12757368

[29] Shi J Zhang R Li J Zhang R . Size profile of cell-free DNA: A beacon guiding the practice and innovation of clinical testing. Theranostics. (2020) 10:4737–48. doi: 10.7150/thno.42565 32308746 PMC7163439

[30] Sanchez C Roch B Thilbault M Blache P Amir A Pastor B . Circulating nuclear DNA structural features, origins, and complete size profile revealed by fragmentomics. JCI Insight. (2021) 6(6):e144561. doi: 10.1172/jci.insight.144561 33571170 PMC8119211

[31] An Y Zhao X Zhang Z Xia Z Yang M Ma L . DNA methylation analysis explores the molecular basis of plasma cell-free DNA fragmentation. Nat Commun. (2023) 14:287. doi: 10.1038/s41467-023-35959-6 36653380 PMC9849216

[32] Han DSC Ni M Chan RWY Wong DKL Hiraki LT Volpi S . Nuclease deficiencies alter plasma cell-free DNA methylation profiles. Genome Res. (2021) 31:2008–21. doi: 10.1101/gr.275426.121 34470801 PMC8559716

[33] Pessei V Macagno M Mariella E Congiusta N Battaglieri V Battuello P . DNA demethylation triggers cell free DNA release in colorectal cancer cells. Genome Med. (2024) 16:118. doi: 10.1186/s13073-024-01386-5 39385243 PMC11462661

[34] Sui W Tan Q Yang M Yan Q Hua L Ou M . Genome-wide analysis of 5-hmC in the peripheral blood of systemic lupus erythematosus patients using an hMeDIP-chip. Int J Mol Med. (2015) 35:1467–79. doi: 10.3892/ijmm.2015.2149 25813249

[35] Pharo H Vedeld HM Sjurgard IV Pinto R Lind GE . From concept to clinic: a roadmap for DNA methylation biomarkers in liquid biopsies. Oncogene. (2025) 44:4814–31. doi: 10.1038/s41388-025-03624-5 41266596 PMC12657234

[36] Sadeh R Sharkia I Fialkoff G Ayelet Rahat Gutin J Chappleboim A . ChIP-seq of plasma cell-free nucleosomes identifies gene expression programs of the cells of origin. Nat Biotechnol. (2021) 39:586–98. doi: 10.1038/s41587-020-00775-6 33432199 PMC7610786

[37] Melamud MM Tolmacheva AS Sizikov AE Klyaus NA Zhuravlev ES Stepanov GA . NETosis-related biomarkers in systemic lupus erythematosus, rheumatoid arthritis, psoriatic arthritis and ankylosing spondylitis: A comparative study. Int J Mol Sci. (2025) 26:12127. doi: 10.3390/ijms262412127 41465552 PMC12733578

[38] Farivar S Shaabanpour Aghamaleki F . Effects of major epigenetic factors on systemic lupus erythematosus. Iran BioMed J. (2018) 22:294–302. doi: 10.29252/ibj.22.5.294 29803202 PMC6058186

[39] Ungerer V Bronkhorst AJ Van den Ackerveken P Herzog M Holdenrieder S . Serial profiling of cell-free DNA and nucleosome histone modifications in cell cultures. Sci Rep. (2021) 11:9460. doi: 10.1038/s41598-021-88866-5 33947882 PMC8096822

[40] Kustanovich A Schwartz R Peretz T Grinshpun A . Life and death of circulating cell-free DNA. Cancer Biol Ther. (2019) 20:1057–67. doi: 10.1080/15384047.2019.1598759 30990132 PMC6606043

[41] Yamamoto R Asano H Tamaki R Saito Y Hosokawa A Watari H . Dynamics and half-life of cell-free DNA after exercise: Insights from a fragment size-specific measurement approach. Diagnostics. (2025) 15:109. doi: 10.3390/diagnostics15010109 39795637 PMC11720216

[42] Tsumita T Iwanaga M . Fate of injected deoxyribonucleic acid in mice. Nature. (1963) 198:1088–9. doi: 10.1038/1981088a0 13994595

[43] Chused TM Steinberg AD Talal N . The clearance and localization of nucleic acids by New Zealand and normal mice. Clin Exp Immunol. (1972) 12:465–76. PMC1553612 4650369

[44] Gauthier VJ Tyler LN Mannik M . Blood clearance kinetics and liver uptake of mononucleosomes in mice. J Immunol. (1996) 156:1151–6. doi: 10.4049/jimmunol.156.3.1151 8557992

[45] Yu SCY Deng J Qiao R Cheng SH Peng W Lau SL . Comparison of single molecule, real-time sequencing and nanopore sequencing for analysis of the size, end-motif, and tissue-of-origin of long cell-free DNA in plasma. Clin Chem. (2022) 69:168–79. doi: 10.1093/clinchem/hvac180 36322427

[46] Jiang P Xie T Ding SC Zhou Z Cheng SH Chan RWY . Detection and characterization of jagged ends of double-stranded DNA in plasma. Genome Res. (2020) 30:1144–53. doi: 10.1101/gr.261396.120 32801148 PMC7462074

[47] Ding SC Lo YMD . Cell-free DNA fragmentomics in liquid biopsy. Diagnostics. (2022) 12:978. doi: 10.3390/diagnostics12040978 35454026 PMC9027801

[48] Ding SC Chan RWY Peng W Huang L Zhou Z Hu X . Jagged ends on multinucleosomal cell free DNA serve as a biomarker for nuclease activity and systemic lupus erythematosus. Clin Chem. (2022) 68:917–26. doi: 10.1093/clinchem/hvac050 35587043

[49] Jiang P Ma MJL Qiao R Shi Y Liu J Zhou Q . Holistic determination of ends of cfDNA molecules. Cell Genom. (2026) 6:101142. doi: 10.1016/j.xgen.2026.101142 41653917 PMC12985390

[50] Chan RWY Serpas L Ni M Volpi S Hiraki LT Tam LS . Plasma DNA profile associated with DNASE1L3 gene mutations: Clinical observations, relationships to nuclease substrate preference, and *in vivo* correction. Am J Hum Genet. (2025) 113(1):234. doi: 10.1016/j.ajhg.2025.12.003 41386233 PMC12824615

[51] Serpas L Chan RJ Jiang P Ni M Sun K Rashidfarrokhi A . Dnase1l3 deletion causes aberrations in length and end-motif frequencies in plasma DNA. PNAS. (2019) 116:641–9. doi: 10.1073/pnas.1815031116 PMC6329986 30593563

[52] Zhou Z Ma MJL Chan RWY Jacky K Peng W Gai W . Fragmentation landscape of cell-free DNA revealed by deconvolutional analysis of end motifs. PNAS. (2023) 120(17):e2220982120. doi: 10.1073/pnas.2220982120 37075072 PMC10151549

[53] Eloranta M-L Rönnblom L . Cause and consequences of the activated type I interferon system in SLE. J Mol Med. (2016) 94:1103–10. doi: 10.1007/s00109-016-1421-4 27094810 PMC5052287

[54] Reizis B . Plasmacytoid dendritic cells: Development, regulation, and function. Immunity. (2019) 50:37–50. doi: 10.1016/j.immuni.2018.12.027 30650380 PMC6342491

[55] Kim HN Montealegre GA Goldbach-Mansky R . Insights from Mendelian interferonopathies: Comparison of CANDLE, SAVI with AGS, monogenic lupus. J Mol Med. (2016) 94:1111–27. doi: 10.1007/s00109-016-1465-5 27678529 PMC5094849

[56] Rönnblom L Leonard D . Interferon pathway in SLE: One key to unlocking the mystery of the disease. Lupus Sci Med. (2019) 6:e000270. doi: 10.1136/lupus-2018-000270 31497305 PMC6703304

[57] Swiecki M Colonna M . The multifaceted biology of plasmacytoid dendritic cells. Nat Rev Immunol. (2015) 15:471–85. doi: 10.1038/nri3865 26160613 PMC4808588

[58] Postal M Vivaldo JF Fernandez-Ruiz R Paredes JL Appenzeller S Niewold TB . Type I interferon in the pathogenesis of systemic lupus erythematosus. Curr Opin Immunol. (2020) 67:87–94. doi: 10.1016/j.coi.2020.10.014 33246136 PMC8054829

[59] Tusseau M Lovšin E Samaille C Pescarmona R Mathieu AL Maggio MC . DNASE1L3 deficiency, new phenotypes, and evidence for a transient type I IFN signaling. J ClinImmunol. (2022) 42:1310–20. doi: 10.1007/s10875-022-01287-5 35670985

[60] Volpi S Angelotti ML Palazzini G Antonelli G Ravaglia F Garibotto F . Lupus nephritis patterns and response to type I interferon in patients with DNASE1L3 variants: Report of three cases. Am J Kidney Dis. (2024) 84:791–7. doi: 10.1053/j.ajkd.2024.05.014 39059688

[61] Soni C Perez OA Voss WN Pucella JN Serpas L Mehl J . Plasmacytoid dendritic cells and type I interferon promote extrafollicular B cell responses to extracellular self-DNA. Immunity. (2020) 52:1022–1038.e7. doi: 10.1016/j.immuni.2020.04.015 32454024 PMC7306002

[62] Tusseau M Khaldi-Plassart S Cognard J Viel S Khoryati L Benezech S . Mendelian causes of autoimmunity: The lupus phenotype. J ClinImmunol. (2024) 44(4):99. doi: 10.1007/s10875-024-01696-8 38619739

[63] Means TK Latz E Hayashi F Murali MR Golenbock DT Luster AD . Human lupus autoantibody– DNA complexes activate DCs through cooperation of CD32 and TLR9. J Clin Invest. (2005) 115:407–17. doi: 10.1172/jci23025 15668740 PMC544604

[64] Hagiwara AM Moore RE Wallace DJ Ishimori M Jefferies CA . Regulation of cGAS-STING pathway - Implications for systemic lupus erythematosus. Rheum Immunol Res. (2021) 2:173–84. doi: 10.2478/rir-2021-0023 36465073 PMC9524788

[65] An J Durcan L Karr RM Briggs TA Rice GI Teal TH . Expression of cyclic GMP‐AMP synthase in patients with systemic lupus erythematosus. Arth Rheum. (2017) 69(4):800–7. doi: 10.1002/art.40002 27863149

[66] Gray EE Treuting PM Woodward JJ Stetson DB . Cutting edge: cGAS is required for lethal autoimmune disease in the Trex1-deficient mouse model of Aicardi–Goutières syndrome. J Immunol. (2015) 195:1939–43. doi: 10.4049/jimmunol.1500969 26223655 PMC4546858

[67] Clancy RM Markham AJ Buyon JP . Endosomal Toll-like receptors in clinically overt and silent autoimmunity. Immunol Rev. (2015) 269:76–84. doi: 10.1111/imr.12383 26683146 PMC4685960

[68] Lech M Anders HJ . The pathogenesis of lupus nephritis. J Am Sic Nephrol. (2013) 24:1357–66. doi: 10.1681/asn.2013010026 23929771 PMC3752952

[69] Sato T Fujii T Yokoyama T Fujita Y Imura Y Yukawa N . Anti-U1 RNP antibodies in cerebrospinal fluid are associated with central neuropsychiatric manifestations in systemic lupus erythematosus and mixed connective tissue disease. Arth Rheum. (2010) 62:3730–40. doi: 10.1002/art.27700 20722023

[70] Goldbach-Mansky R Alehashemi SA . Emerging concepts and treatments in autoinflammatory interferonopathies and monogenic systemic lupus erythematosus. Nat Rev Rheum. (2024) 21:22–4. doi: 10.1038/s41584-024-01184-8 39623155

[71] Furie RA Morand EF Bruce IN Manzi S Kalunian KC Vital EM . Type I interferon inhibitor anifrolumab in active systemic lupus erythematosus (TULIP-1): A randomised, controlled, phase 3 trial. Lancet Rheumatol. (2019) 1:e208–19. doi: 10.1016/s2665-9913(19)30076-1 38229377

[72] Furie R Khamashta M Merrill JT Werth VP Kalunian K Brohawn P . Anifrolumab, an anti-interferon-α receptor monoclonal antibody, in moderate-to-severe systemic lupus erythematosus. Arth Rheum. (2017) 69(2):376–86. doi: 10.1016/j.berh.2017.10.005 28130918 PMC5299497

[73] Khamashta M Merrill JT Werth VP Furie R Kalunian K Illei GG . Sifalimumab, an anti-interferon-α monoclonal antibody, in moderate to severe systemic lupus erythematosus: A randomised, double-blind, placebo-controlled study. Ann Rheum Dis. (2016) 75:1909–16. doi: 10.1136/annrheumdis-2015-208562 27009916 PMC5099191

[74] Deligeorgakis D Skouvaklidou E Adamichou C . Interferon inhibition in SLE: From bench to bedside. Med J Rheum. (2024) 35:354–. doi: 10.31138/mjr.010324.iis 39193183 PMC11345605

[75] Martin G . Novel therapies in plaque psoriasis: A review of tyrosine kinase 2 inhibitors. Dermat Ther. (2023) 13:417–35. doi: 10.1007/s13555-022-00878-9 36592300 PMC9884727

[76] Karnell JL Wu Y Mittereder N Smith MA Gunsior M Yan L . Depleting plasmacytoid dendritic cells reduces local type I interferon responses and disease activity in patients with cutaneous lupus. Sci Trans Med. (2021) 13:eabf8442. doi: 10.1126/scitranslmed.abf8442 34039741

[77] Carrington EM Zhang JG Sutherland RM Vikstrom IB Brady JL Soo P . Prosurvival Bcl-2 family members reveal a distinct apoptotic identity between conventional and plasmacytoid dendritic cells. PNAS. (2015) 112:4044–9. doi: 10.1073/pnas.1417620112 25775525 PMC4386329

[78] Liu P Zhao L Zitvogel L Kepp O Kroemer G . The BCL2 inhibitor venetoclax mediates anticancer effects through dendritic cell activation. Cell Death Differ. (2023) 30:2447–51. doi: 10.1038/s41418-023-01232-y 37845384 PMC10733328

[79] Peng X Tang F Li Y Bai J Li L Zhang L . Combination of BCL-2 inhibitors and immunotherapy: A promising therapeutic strategy for hematological Malignancies. Discov Oncol. (2024) 15:311. doi: 10.1007/s12672-024-01161-3 39060763 PMC11282050

[80] Rowland SL Riggs JM Gilfillan S Bugatti M Vermi W Kolbeck R . Early, transient depletion of plasmacytoid dendritic cells ameliorates autoimmunity in a lupus model. J Exp Med. (2014) 211:1977–91. doi: 10.1084/jem.20132620 25180065 PMC4172228

[81] Abdalla MA Elmofty SA Elmaghraby AA Khalifa RH . Antinucleosome antibodies in systemic lupus erythematosus patients: Relation to anti-double stranded deoxyribonucleic acid and disease activity. Egyp J Rheumatol. (2017) 40:29–33. doi: 10.1016/j.ejr.2017.05.004 38826717

[82] Hartl J Serpas L Wang Y Rashidfarrokhi A Perez OA Sally B . Autoantibody-mediated impairment of DNASE1L3 activity in sporadic systemic lupus erythematosus. J Exp Med. (2021) 218(5):e20201138. doi: 10.1136/lupus-2021-lupus21century.39 33783474 PMC8020718

[83] Groff K Brown J Clippinger AJ . Modern affinity reagents: Recombinant antibodies and aptamers. Biotechnol Adv. (2015) 33:1787–98. doi: 10.1016/j.bioteChadv.2015.10.004 26482034

[84] Gomez-Bañuelos E Yu Y Li J Cashman KS Paz M Trejo-Zambrano MI . Affinity maturation generates pathogenic antibodies with dual reactivity to DNase1L3 and dsDNA in systemic lupus erythematosus. Nat Commun. (2023) 14(1):1388. doi: 10.1038/s41467-023-37083-x 36941260 PMC10027674

[85] Bruschi M Angeletti A Kajana X Moroni G Sinico RA Fredi M . Evidence for charge-based mimicry in anti dsDNA antibody generation. J Autoimmun. (2022) 132:102900. doi: 10.1016/j.jaut.2022.102900 36087539

[86] Han BK Wysham KD Cain KC Tyden H Bengtsson AA . Neutrophil and lymphocyte counts are associated with different immunopathological mechanisms in systemic lupus erythematosus. Lupus Sci Med. (2020) 7:e000382. doi: 10.1136/lupus-2020-000382 32444416 PMC7247402

[87] Baker T Sharifian H Newcombe PJ Gavin PG Lazarus MN Ramaswamy M . Type I interferon blockade with anifrolumab in patients with systemic lupus erythematosus modulates key immunopathological pathways in a gene expression and proteomic analysis of two phase 3 trials. Ann Rheum Dis. (2024) 83:1018–27. doi: 10.1136/ard-2023-225445 38569851 PMC12056589

[88] ClinicalTrials.gov . A phase 1 randomized, placebo-controlled, blinded, multiple ascending dose study to evaluate VIB7734 in systemic lupus erythematosus, cutaneous lupus erythematosus, Sjögren's syndrome, systemic sclerosis, polymyositis, and dermatomyositis. (2020). Identifier NCT03817424. Available online at: https://clinicaltrials.gov/study/NCT03817424 (Accessed April 9, 2026).

[89] Sprow G Dan J Merola JF Werth VP . Emerging therapies in cutaneous lupus erythematosus. Front Med. (2022) 9. doi: 10.3389/fmed.2022.968323 35899214 PMC9313535

[90] Jarzebska N Rodionov RN Voit-Bak K Straube R Mücke A Tselmin S . Neutrophil extracellular traps (NETs) as a potential target for anti-aging: Role of therapeutic apheresis. Horm Metabol Res. (2025) 57(11):632–8. doi: 10.1055/a-2444-3422 39788160 PMC12638183

[91] Joy A Muralidharan A Alfaraj M Shantharam D Cherukuri ASS Muthukumar A . The role of belimumab in systemic lupus erythematosis: A systematic review. Cureus. (2022) 14(6):e25887. doi: 10.7759/cureus.25887 35844357 PMC9277571

